# Heterozygous factor V Leiden mutation manifesting with combined central retinal vein occlusion, cilioretinal artery occlusion, branch retinal artery occlusion, and anterior ischaemic optic neuropathy: a case report

**DOI:** 10.1186/s12886-022-02278-1

**Published:** 2022-02-05

**Authors:** Anis Mahmoud, Molka Khairallah, Hassen Ibn Hadj Amor, Mohamed Habib Lahdhiri, Nesrine Abroug, Riadh Messaoud, Moncef Khairallah

**Affiliations:** 1 Department of Ophthalmology, Tahar Sfar University Hospital, Mahdia, Faculty of Medicine, University of Monastir, Monastir, Tunisia; 2 Department of Ophthalmology, Fattouma Bourguiba University Hospital, Faculty of Medicine, University of Monastir, Monastir, Tunisia; 3 Department of Cardiology, Tahar Sfar University Hospital, Mahdia, Faculty of Medicine, University of Monastir, Monastir, Tunisia

**Keywords:** Central retinal vein occlusion, Cilioretinal artery occlusion, Anterior ischaemic optic neuropathy, Factor V Leiden mutation, Case report

## Abstract

**Background:**

Our purpose was to describe a patient who developed combined central retinal vein occlusion (CRVO), cilioretinal artery occlusion, branch retinal artery occlusion (BRAO), and anterior ischaemic optic neuropathy (AION) followed by CRVO in the second eye because of the heterozygous factor V Leiden (FVL) mutation.

**Case presentation:**

A 39-year-old female with a history of recurrent pregnancy losses presented with acute blurred vision in the right eye (RE), with visual acuity limited to counting fingers. She was diagnosed with combined impending CRVO, cilioretinal artery occlusion, BRAO, and AION. The results of thrombophilia testing, not including the FVL mutation, were negative. Retinal atrophy with vascular attenuation and optic disc pallor developed after resolution of acute retinal findings. Nine months after initial presentation, the patient developed an impending CRVO in the left eye (LE), with a secondary progression to a complete CRVO causing a decrease in best corrected visual acuity (BCVA) to 20/40. The patient was determined to be heterozygous for the FVL mutation. She subsequently was treated with acenocoumarol. At the last follow-up visit, the BCVA was 20/400 in the RE and 20/20 in the LE, and there was a complete resolution of the acute CRVO findings in the LE.

**Conclusion:**

Our case shows that the heterozygous FVL mutation may manifest with combined retinal vascular occlusion involving multiple sites in both eyes. Early recognition of such an inherited thrombophilic disorder is important because it implies the need for long-term anticoagulative therapy to reduce the patient’s risk of recurrent, sight-threatening and life-threatening thrombotic events.

## Introduction

Ocular vaso-occlusive disease usually occurs in elderly patients with identifiable risk factors, such as systemic hypertension, diabetes mellitus, hyperlipidaemia, history of smoking, and atherosclerosis [1]. It may also be caused by inherited or acquired thrombophilic disorders, including hyperhomocysteinemia, the factor V Leiden (FVL) mutation, the prothrombin G20210A mutation, antithrombin III deficiency, protein C deficiency, protein S deficiency, and the lupus anticoagulant found in antiphospholipid syndrome. The FVL mutation is a point mutation in the factor V gene in which glutamine is substituted for arginine at position 506. This leads to an increased risk of thrombosis through resistance to activated protein C (APC-R). The FVL mutation was found to be the most common inherited thrombophilic disorder in the Caucasian population, accounting for up to 37% of venous thrombosis cases. The homozygous FVL mutation form carries a higher risk of thromboembolism than the heterozygous form [1].

The FVL mutation has been associated with retinal vein occlusion, mainly in young patients without identifiable cardiovascular risk factors [2]. The association of FVL with retinal artery occlusion, ischaemic optic neuropathy, or combined vascular occlusions has been scarcely described [2–5]. We herein describe a case of the FVL mutation manifesting with CRVO associated with unilateral cilioretinal artery occlusion, BRAO, and ischaemic optic neuropathy followed by CRVO in the second eye.

## Case presentation

A 39-year-old female with a history of recurrent pregnancy losses for which investigation was not available presented with acute vision loss in the RE.

**Fig. 1:**
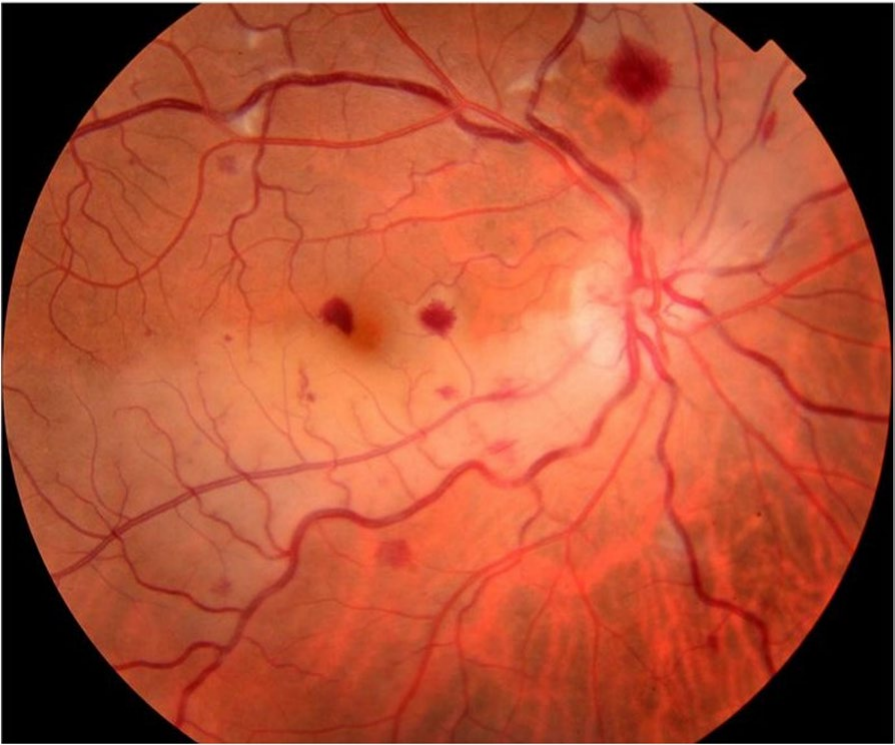
Fundus photograph of the right eye shows a few superficial and deep intraretinal haemorrhages, cotton-wool spots, and dilated tortuous veins. There was also optic disc swelling and ischaemic retinal whitening in the posterior pole inferotemporally along a cilioretinal artery and in the superonasal quadrant

**Fig. 2:**
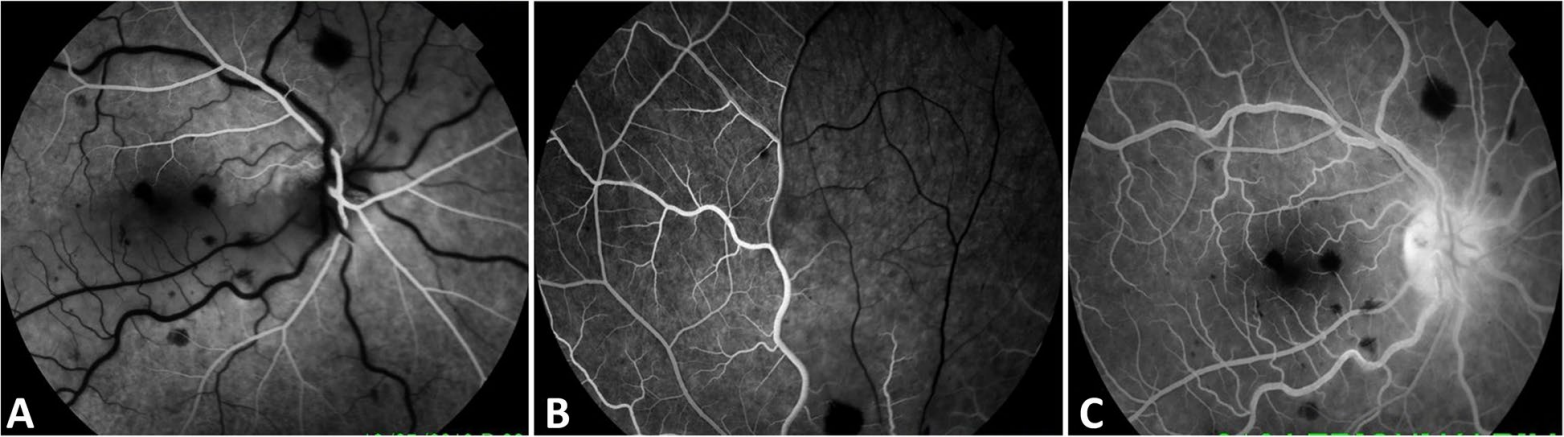
Fluorescein angiography of the right eye shows delayed filling of the cilioretinal artery (**A**) and of the superonasal branch retinal artery (**B**). Also note the filling defect in the inferior part of the optic disc at the early phase (**A**) and the late staining at the late phase (**C**)

**Fig. 3:**
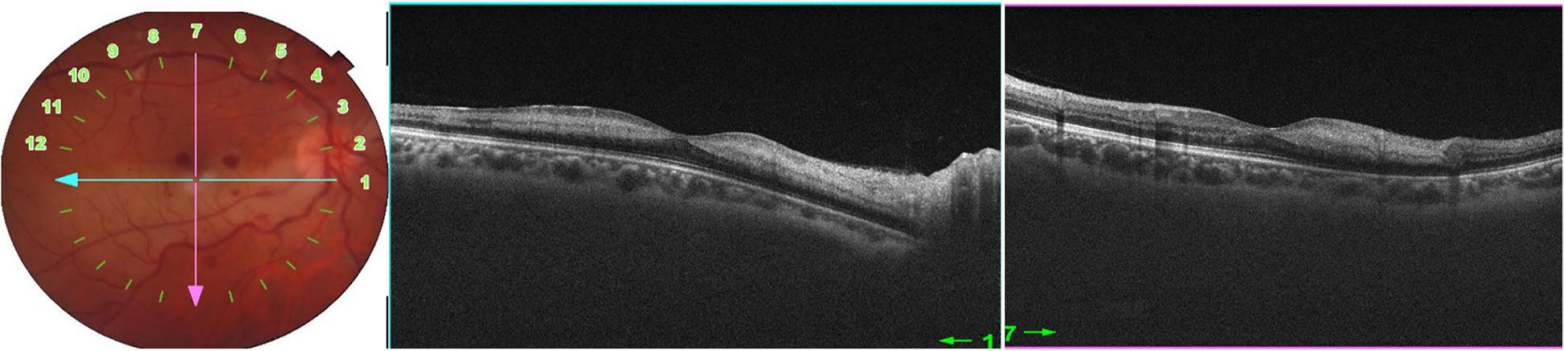
Macular SS-OCT scan through the area of retinal whitening shows hyperreflective band-like lesions in the middle retina

On examination, BCVA was counting fingers, with associated relative afferent pupillary defect, in the RE and 20/20 in the LE. Slit-lamp examination showed quiet anterior segment and vitreous and a normal intraocular pressure in both eyes. Funduscopic examination of the RE revealed a few superficial and deep intraretinal haemorrhages, cotton-wool spots, and dilated tortuous veins. There was also optic disc swelling and ischaemic retinal whitening in the posterior pole inferotemporally along a cilioretinal artery and in the superonasal quadrant (Fig. 1). The LE fundus was normal. Fluorescein angiography of the RE showed delayed filling of the cilioretinal artery and of the superonasal branch retinal artery (Fig. 2A, B). There was also a filling defect in the inferior part of the optic disc at the early phase, with late staining at the late phase (Fig. 2A, C). Swept source optical coherence tomography (SS-OCT) showed perifoveal band-like hyperreflective lesions at the level of the inner nuclear layer corresponding to retinal whitening seen clinically (Fig. 3). Imaging findings in the LE were unremarkable. Clinical and multimodal imaging in the RE were consistent with a diagnosis of combined impending CRVO, cilioretinal artery occlusion, BRAO, and AION.

The results of physical examination, cardiologic evaluation, including carotid artery echocardiography, and standard laboratory investigations were normal or negative. The results of the hypercoagulability workup, including prothrombin time/partial prothrombin time (PT/PTT), plasma homocysteine, antiphospholipid antibodies (lupus anticoagulant, anticardiolipin), protein C, protein S, the prothrombin G20210A mutation, and antithrombin, were negative. However, testing for FVL mutations, which was not routinely available in our hospital, was not performed in our patient at initial presentation. The patient was treated with acetylsalicylic acid.

**Fig. 4:**
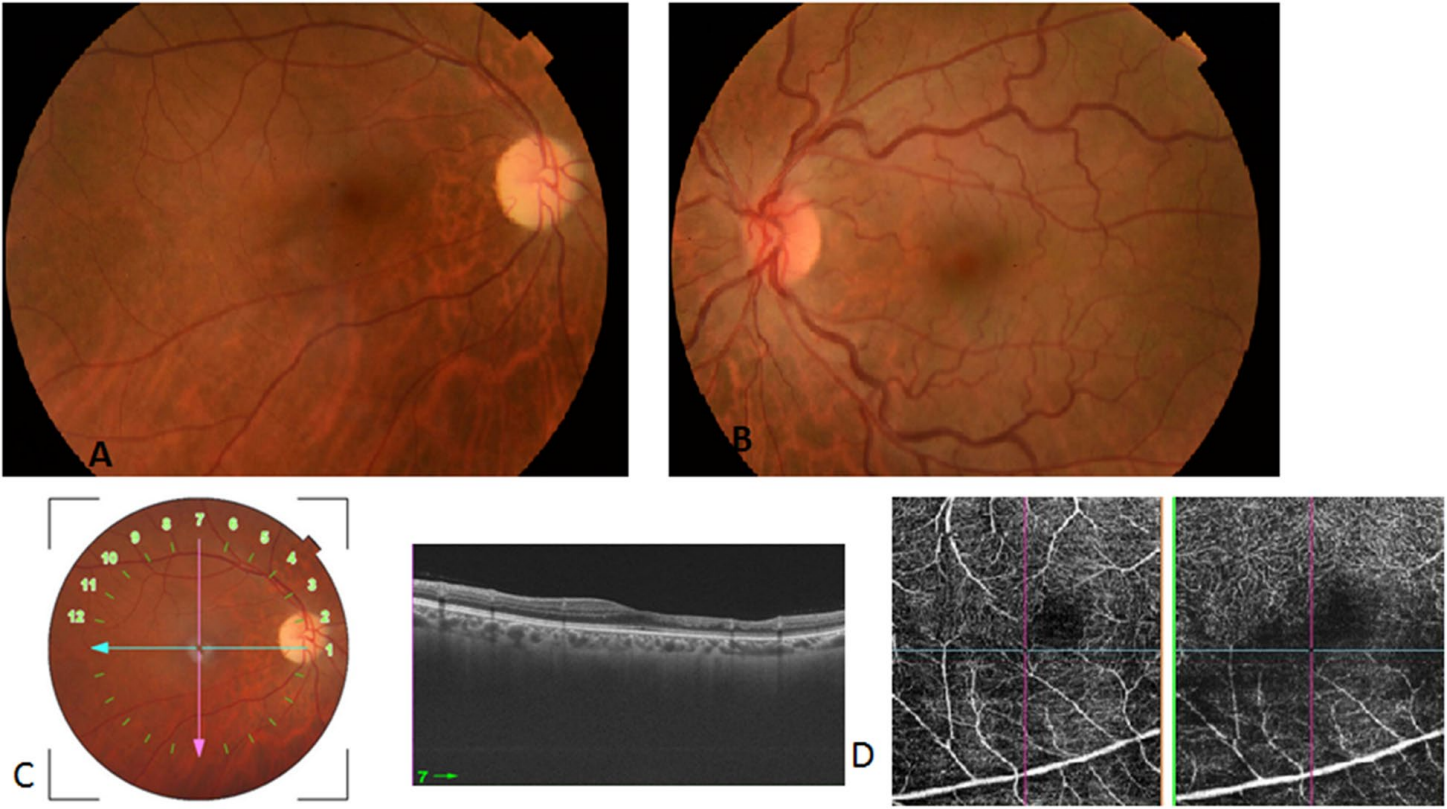
**A** Fundus photograph of the RE nine months after initial presentation shows the presence of diffuse optic disc pallor and the resolution of acute retinal changes with diffuse attenuation of the cilioretinal artery and retinal arterioles. **B** Fundus photograph of the LE shows diffuse tortuosity and dilatation of retinal veins consistent with impending CRVO. **C** Vertical macular SS-OCT scan of the RE shows thinning and atrophy of the inner nuclear layer. **D** OCT angiography of the RE shows marked vascular alterations involving the deep capillary plexus with significant projection artefacts from the superficial vascular plexus on the attenuated deep retinal capillary plexus

Nine months later, she presented with mild blurry vision in the LE. Visual acuity was 20/400 in the RE and 20/25 in the LE. The anterior segment and intraocular pressure were normal in both eyes. Fundus examination of the RE showed the presence of diffuse optic disc pallor and the resolution of acute retinal changes with diffuse attenuation of the cilioretinal artery and retinal arterioles (Fig. 4A). In the LE, there was diffuse tortuosity and dilatation of retinal veins consistent with impending CRVO (Fig. 4B).

SS-OCT of the RE showed thinning and atrophy of the inner nuclear layer (Fig. 4C). SS-OCTA of the RE showed marked vascular alterations with visibility of superficial retinal vessels seen as projection artefacts on the attenuated deep retinal capillary plexus (Fig. 4D).

**Fig. 5:**
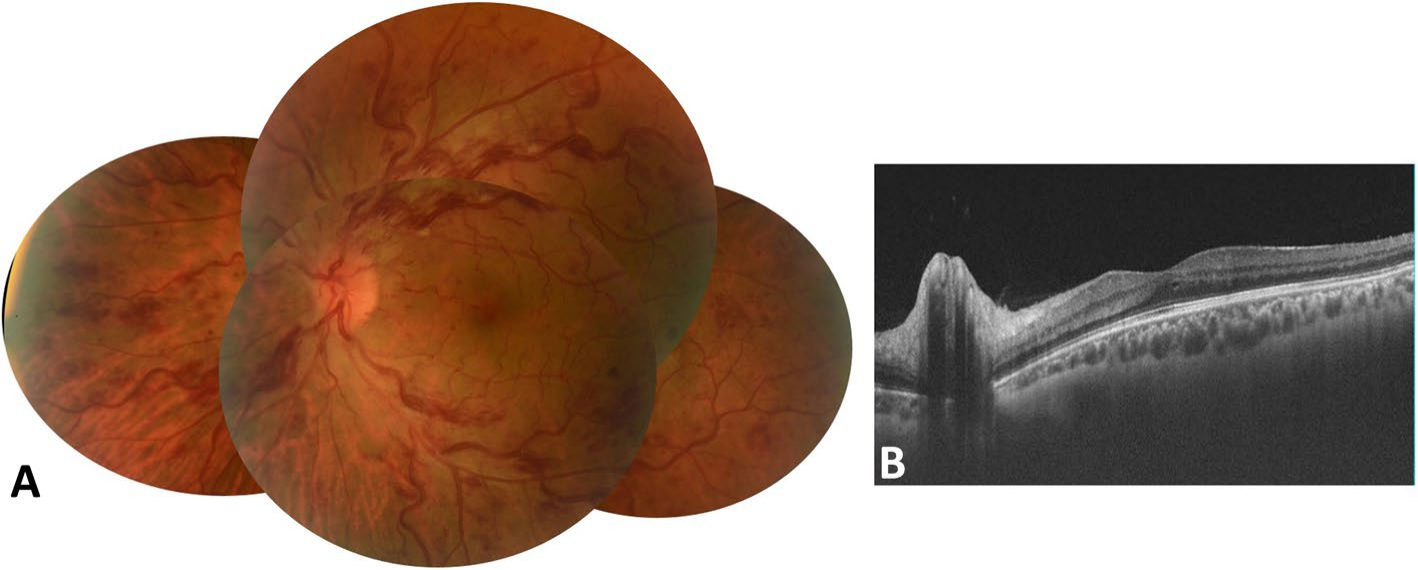
Eight weeks later, impending CRVO of the LE progressed to complete CRVO (**A**) with associated moderate central macular thickening on SS-OCT (**B**)

**Fig. 6:**
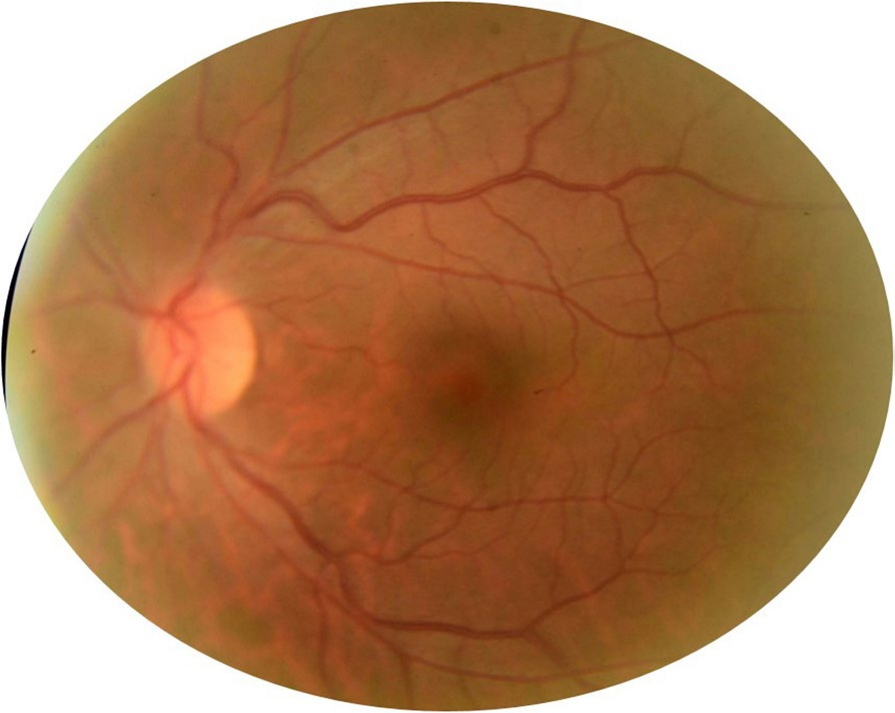
Fundus photograph of the LE shows complete resolution of the acute CRVO findings in the LE

Eight weeks later, the visual acuity of the LE was 20/40, and the impending CRVO had progressed to complete CRVO (Fig. 5). A repeated hypercoagulability workup revealed abnormal resistance to activated protein C, and the patient was then tested for the FVL mutation. The patient was found to be heterozygous for the FVL mutation. The patient was treated with acenocoumarol. At the last follow-up visit, the BCVA was 20/400 in the RE and 20/20 in the LE. Fundus examination of the LE showed resolution of retinal haemorrhages and retinal vein tortuosity and dilatation (Fig. 6). There were no further changes in the right fundus.

## Discussion and conclusions

This is a unique case of the FVL mutation manifesting with sequential bilateral ocular vaso-occlusive disease involving multiple sites documented with multimodal imaging, including CRVO, cilioretinal artery occlusion, BRAO, and AION. In this young female with a history of recurrent pregnancy losses, an initial clinical and laboratory evaluation allowed us to rule out any underlying cardiovascular disease, inflammatory condition, ocular compression, trauma, or thrombophilic disorder other than FVL. Notably, repeated, more comprehensive thrombophilia testing allowed for the detection of an underlying heterozygous FVL mutation, and the patient subsequently received anticoagulant therapy.

FVL heterozygosity is found in 30% of women with unexplained recurrent pregnancy loss compared with 1–10% of controls. It is also associated with other complications, such as preeclampsia [6]. The FVL mutation is characterized by an increased risk for venous thromboembolism (VTE). Deep venous thrombosis and pulmonary embolism are the most common manifestations. Other unusual locations can also occur. There is a lack of clarity regarding when the tests for FVL should be performed. A recent systematic review and meta-analysis data do not support routine thrombophilia screening in the diagnostic workup of all patients presenting with retinal vascular occlusion [7]. FVL is not recommended as a routine initial test. However, testing may be considered in individuals younger than 50 years of age with unexplained arterial thrombosis, in cases following a first-time unprovoked VTE, in patients with a history of recurrent VTE, in women with repeated miscarriages, in patients with a family history of thromboembolism and in patients with atypical or combined ocular vascular occlusion [8].

The FVL mutation can be diagnosed by a functional resistance to APC-R assay or by genetic testing using DNA-based methods for the detection of the FVL mutation and for the distinction between heterozygotes and homozygotes. Genetic testing methods primarily include PCR amplification of the defect region from genomic material followed by restriction enzyme cleavage [9]. Early diagnosis is of utmost importance for timely initiation of anticoagulant treatment to prevent further potentially sight-threatening or life-threatening thrombotic events**.** According to standard guidelines, the initial treatment consists of low molecular weight heparin, followed by vitamin K antagonists. A target international normalized ratio (INR) of 2.5 has been proven to provide effective anticoagulation [8]. The duration of oral anticoagulation therapy is, in general, 3 to 6 months, based on the risk of recurrence and bleeding complications.

## Data Availability

The datasets used and/or analysed during the current study are available from the corresponding author on reasonable request.

## Abbreviations

AION: Anterior Ischaemic Optic Neuropathy
APC-R: Resistance to Activated Protein C
BCVA: Best Corrected Visual Acuity
BRAO: Branch Retinal Artery Occlusion
CRVO: Central Retinal Vein Occlusion
FVL: Factor V Leiden
LE: Left Eye
PCR: Polymerase Chain Reaction
RE: Right Eye
SS-OCT: Swept Source Optical Coherence Tomography
VTE: Venous ThromboEmbolism
